# PTPN14 promotes gastric cancer progression by PI3KA/AKT/mTOR pathway

**DOI:** 10.1038/s41419-023-05712-4

**Published:** 2023-03-10

**Authors:** Hui Li, Bingxin Guan, Sen Liu, Haiting Liu, Lin Song, Guohao Zhang, Ruinan Zhao, Chengjun Zhou, Peng Gao

**Affiliations:** 1 Key Laboratory for Experimental Teratology of Ministry of Education, Department of Pathology, School of Basic Medical Sciences, Shandong University, 250012 Jinan, Shandong China; 2 Department of Pathology, Qilu Hospital, Shandong University, 250012 Jinan, Shandong China; 3 Department of Pathology, The Second Hospital of Shandong University, 250012 Jinan, Shandong China; 4 Department of Pathology, Shandong Provincial Hospital Affiliated to Shandong First Medical University, 250012 Jinan, Shandong China

**Keywords:** Gastric cancer, Tumour biomarkers, Oncogenesis

## Abstract

Gastric cancer is a high molecular heterogeneous disease with a poor prognosis. Although gastric cancer is a hot area of medical research, the mechanism of gastric cancer occurrence and development is still unclear. New strategies for treating gastric cancer need to be further explored. Protein tyrosine phosphatases play vital roles in cancer. A growing stream of studies shows that strategies or inhibitors targeting protein tyrosine phosphatases have been developed. PTPN14 belongs to the protein tyrosine phosphatase subfamily. As an inert phosphatase, PTPN14 has very poor activity and mainly functions as a binding protein through its FERM (four-point-one, ezrin, radixin, and moesin) domain or PPxY motif. The online database indicated that PTPN14 may be a poor prognostic factor for gastric cancer. However, the function and underlying mechanism of PTPN14 in gastric cancer remain unclear. We collected gastric cancer tissues and detected the expression of PTPN14. We found that PTPN14 was elevated in gastric cancer. Further correlation analysis indicated that PTPN14 was relevant with the T stage and cTNM (clinical tumor node metastasis classification) stage. The survival curve analysis showed that gastric cancer patients with higher PTPN14 expression had a shorter survival time. In addition, we illustrated that CEBP/β (CCAAT enhanced binding protein beta) could transcriptionally activate PTPN14 expression in gastric cancer. The highly expressed PTPN14 combined with NFkB (nuclear factor Kappa B) through its FERM domain and accelerated NFkB nucleus translocation. Then, NFkB promoted the transcription of PI3KA and initiated the PI3KA/AKT/mTOR pathway to promote gastric cancer cell proliferation, migration, and invasion. Finally, we established mice models to validate the function and the molecular mechanism of PTPN14 in gastric cancer. In summary, our results illustrated the function of PTPN14 in gastric cancer and demonstrated the potential mechanisms. Our findings provide a theoretical basis to better understand the occurrence and development of gastric cancer.

## Background

Globally, gastric cancer is the fourth cause of cancer-related deaths and the fifth most common cancer [1]. Although there are several effective therapeutics for gastric cancer, the overall survival rate of gastric cancer is still very poor. For this reason, we need to further explore the mechanism of gastric cancer occurrence and development.

Recent research has shown that protein tyrosine phosphatases play dual roles in cancer [2, 3]. Most of the phosphatases mainly functioned as tumor suppressors by dephosphorylating oncogenic substrates, such as PTPN12 [4]. Whereas some phosphatases acted as oncogenes in cancer to promote cancer progression. PTPN1 activated the MAPK/ERK and PI3K/AKT pathways to promote glioma progression [5]. PTPN21 was highly expressed in B cell non-Hodgkin’s gastric lymphoma and promoted the activity of STAT5 [6]. Therefore, the roles of phosphatases in cancer are very important and worth studying.

PTPN14 is an inert protein tyrosine phosphatase, which is composed of the FERM domain, the protein tyrosine phosphatase (PTP) domain, and the middle region [7, 8]. Although PTPN14 has very poor enzymatic activity [9], it plays important functions by dephosphorylating substrate proteins such as YAP, β-catenin, p130Cas, RIN1, PKC-δ, and CAV1 [10–14]. The FERM domain mediates the interaction of PTPN14 with other proteins. Interestingly, PTPN14 could translocate to the nucleus and may function in cell proliferation [15]. In rheumatoid arthritis, PTPN14 promoted the TGFβ pathway [16]. Besides, PTPN14 promoted the nuclear factor Kappa B (NFkB) pathway in innate immune regulation [17]. The online database indicated that PTPN14 may be related to the prognosis of gastric cancer patients. However, there are very few studies illustrating the function of PTPN14 in gastric cancer. In this research, we combined sample validation, cellular, and molecular approaches to explore the function of PTPN14 in gastric cancer. Sample validation revealed that PTPN14 was highly expressed in gastric cancer, especially with lymph node metastasis (LNM). Further research indicated that PTPN14 promoted gastric cancer cell proliferation, migration, and invasion via the FERM domain. The FERM domain of PTPN14 was shown to bind with NFkB, promoting NFkB nucleus translocation. As a result, the PI3KA/AKT/mTOR pathway was activated subsequently. In conclusion, PTPN14 may play as a tumor promoter in gastric cancer.

## Methods

### Tissue samples

The gastric samples included 426 cases of paraffin-embedded tissues from 2013 to 2017 in the Second Hospital of Shandong University. This research was approved by the Research Ethics Committee of the Shandong University of Medicine and was in accordance with the ethical guidelines of the World Medical Association Declaration of Helsinki.

### Immunohistochemistry (IHC)

The gastric cancer samples and paired normal gastric tissues were gathered into a gastric tissue microarray. The samples were incubated with PTPN14 antibody (1:200 dilution, Abcam, Cambridge, UK, ab204321). The intensity of IHC staining and the proportion were evaluated by two senior pathologists independently. The intensity of IHC staining was graded as follows: 0 (negative, no brown color), 1 (weak, light brown), 2 (moderate, brown), and 3 (strong, deep brown). The proportion of staining was graded as follows: 0 (negative), 1 (<33%), 2 (33–66%), and 3 (>66%). Staining scores were calculated as follows: score (maximum of 9) = staining intensity × staining proportion [18]. The median score was chosen as the cut-off value to separate the PTPN14 low expression group and PTPN14 high expression groups.

### Cell culture and cell transfection

Human gastric cancer cell lines AGS, BGC-823, HGC-27, MKN-45, and SGC-7901 were obtained from the American Type Culture Collection. These gastric cancer cell lines were cultured with RPMI-1640 medium, supplemented with 10% fetal bovine serum (Gibco BRL, Grand Island, NY, U.S.) and 1% Penicillin–Streptomycin Solution (Gibco BRL, Grand Island, USA).

For transient transfection, we used Neofect DNA transfection reagent (Neofect Biotech Co., Ltd, Beijing, China; TF20121201) following the instructions.

For stable transfection, we ordered lentivirus from Jikai Gene company, Shanghai, China. We transformed cells with lentivirus according to the instructions and selected with puromycin.

### Real-time quantitative polymerase chain reaction (RT-qPCR)

We used the reverse transcriptase cDNA synthesis kit (Toyobo, Shanghai, China) following the instructions. We detected the mRNA expression level of PTPN14, PI3KA, CDK4, KIF11, TACC3, and PI3KD using SYBR Green Real-time PCR Master Mix (Roche, Mannheim, Germany). The primer sequences are shown in Supplemental Table 1.

### Cell proliferation, migration, and invasion assays

Cell proliferation (CCK-8 and EDU) assays and transwell assays were performed as previously described [19, 20].

### Identification of PTPN14 core promoter region

The 2000-bp transcription start site upstream sequence of PTPN14 was obtained from the UCSC Genome Browser (http://genome.ucsc.edu/). The putative PTPN14 promoter sequences (−2000/0, −1000/0, −500/0, −250/0, −125/0) subcloned into pGL3 were ordered from the General biology company, Chuzhou, China. We used the Dual-Luciferase Reporter system (Promega, Madison, USA) to detect the luciferase activity.

### Chromatin immunoprecipitation (ChIP) assay

The CHIP assay was performed as previously described [20]. The DNA fragments precipitated by anti-CEBP/β antibody (Abmart, Shanghai, China, T55276) or anti-IgG antibody were quantified using qPCR. The primer sequences are shown in Supplemental Table 1.

### Western blot and co-immunoprecipitation (Co-IP) assay

The western blot and Co-IP assays were performed as previously described [19, 20]. The western blot bands were quantified by the Image J software. The following primary antibodies were used for western blot in this research, including PTPN14 (Abcam, Cambridge, UK, ab204321), CEBP/β (Abmart, Shanghai, China, T55276), Flag (Cell Signaling Technology, Boston, USA, 14793), GADPH (Abmart, Shanghai, China, M20006), PI3KA (Abcam, Cambridge, UK, ab40776), PI3KD (Abcam, Cambridge, UK, ab109006), AKT (Invitrogen, CA, USA, 44–609G), p-AKT-S473 (Invitrogen, CA, USA, PA5–104445), mTOR (Invitrogen, CA, USA, PA1–518), p-mTOR-S2448 (Invitrogen, CA, USA, 44–1125G), CDK4 (Cell Signaling Technology, Boston, USA, 12790), KIF11 (Abmart, Shanghai, China, PH2307), TACC3 (Abmart, Shanghai, China, T55133), and NFkB (p65 subunit, Abmart, Shanghai, China, MA9200).

### NFkB inhibitor assays

PTD- p65-P1 Peptide TFA (MCE, NJ, USA, HY-P1832A) is a NFkB inhibitor [21]. We incubated cells with TFA with a concentration of 150 μM for 12 h. The proliferation, migration, and invasion assays were performed as previously described [19, 20].

### Mice models

Six-week-old male BALB/c nude mice were ordered from Weitong Lihua Biotechnology (Beijing, China). In all, 5 × 10^5^ BGC-823 cells stably transfected with LV-PTPN14 or LV-NC were subcutaneously injected into the armpit (*n* = 6, random allocation) or tail vein injected into the mice (*n* = 6, random allocation). The tumor growth was recorded every 3 days and the tumor volume was calculated as *V* = (length × width^2^)/2 [19]. Three weeks after injection, the mice were under anesthesia and then sacrificed to isolate tumors or lungs. The xenograft tumors were cut into two parts. One half was preserved in liquid nitrogen as fresh tissues, and the other half was prepared as paraffin-embedded tissues. The fresh tissues were used for immunofluorescence, RT-qPCR, and western blot. The paraffin-embedded tissues were prepared into slices. The hematoxylin–eosin staining of slices was performed to observe the morphological changes of cells. The IHC staining of slices was taken to verify the expression of PTPN14. All the animal experiment operations were approved by the Committee on the Ethics of Animal Experiments of Basic Medical Sciences, Shandong University.

### Data analysis

The statistical analysis was carried out using GraphPad Prism 8.0 (GraphPad Software, USA). The Student’s *t* test was used for analyzing the difference between the two groups. One-way analysis of variance was used for multiple groups. Pearson’s chi-squared test was used for correlation analysis. Three independent repeated experiments were performed, except for sample tissue validation and nude mice experiments.

## Results

### PTPN14 is upregulated and related to prognosis in gastric cancer

**Fig. 1:**
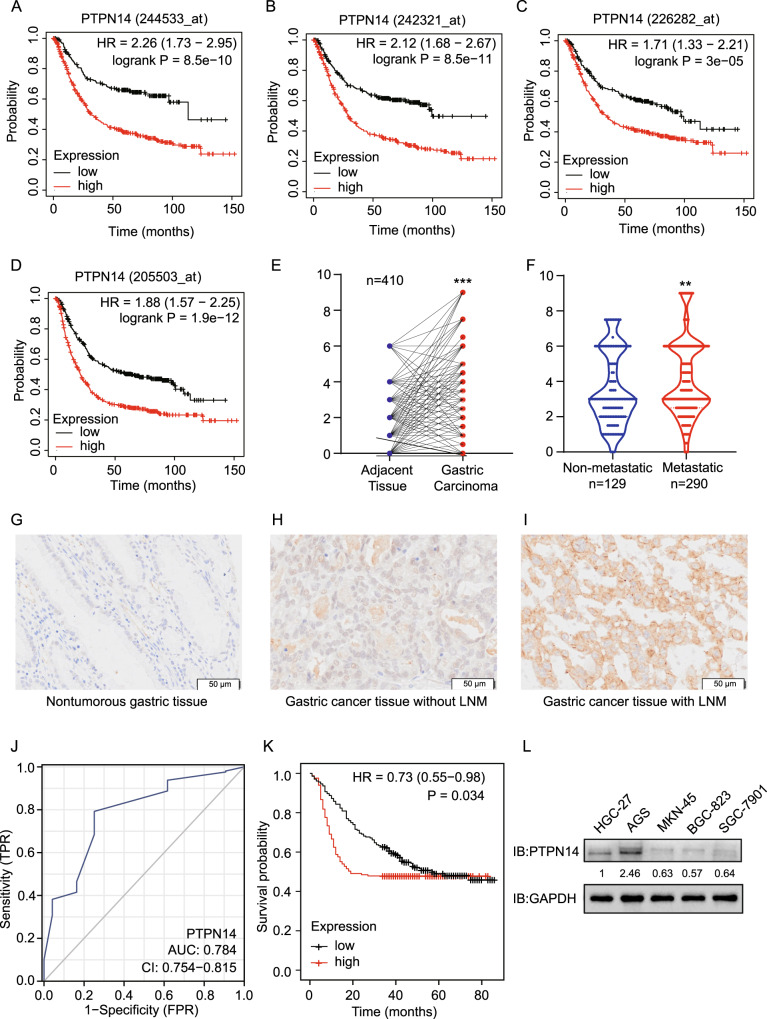
PTPN14 is an adverse factor in gastric cancer. **A**–**D** In the Kaplan–Meier Plotter online database, the gastric cancer patients with higher PTPN14 expression had a shorter relapse-free survival time. The desired Affy IDs are valid: 244533_at (PTPN14) (**A**), 242321_at (PTPN14) (**B**), 226282_at (PTPN14) (**C**), 205503_at (PTPN14) (**D**). **E** The expression of PTPN14 in gastric cancer tissues was higher than in the paired adjacent tissues (*n* = 410). ***p < 0.005; gastric cancer tissues were compared with adjacent normal tissues. **F** The expression of PTPN14 was higher in gastric cancer tissues with LNM (lymph node metastasis) than in gastric cancer tissues without LNM. **p < 0.01; gastric cancer tissues with LNM were compared with gastric cancer tissues without LNM. **G**–**I** The PTPN14 expression was verified by immunohistochemistry in gastric tissues. PTPN14 was mainly expressed in the cytoplasm. PTPN14 is hardly expressed in normal gastric tissues (**G**), weak staining with a low proportion in gastric cancer tissues without LNM (H), and strong staining with a high proportion in gastric cancer tissues with LNM (**I**). **J** The ROC curve illustrated that the PTPN14 expression level could distinguish gastric cancer tissues from normal gastric tissues. **K** The overall survival curve of gastric cancer patients indicated that PTPN14 was an adverse factor. *p* = 0.034; gastric cancer patients with high PTPN14 expression were compared with gastric cancer patients with low PTPN14 expression. **L** Western blot showed the expression of PTPN14 in five gastric cancer cell lines. PTPN14 was highly expressed in HGC-27 and AGS cell lines. PTPN14 was lowly expressed in MKN-45, BGC-823, and SGC-7901 cell lines.

At present, the research on PTPN14 in gastric cancer is rarely reported and remains to be further explored. The Kaplan–Meier Plotter online database indicated that PTPN14 expression level was related to gastric cancer prognosis. Gastric cancer patients with higher PTPN14 expression obtained a significantly shorter relapse-free survival time than patients with lower PTPN14 expression (Fig. 1A–D). We, therefore, collected 426 gastric cancer samples and adjacent normal tissues from 2013 to 2017 in the Second Hospital of Shandong University (Supplemental Fig. 1A, B). Immunohistochemistry analysis showed that the PTPN14 expression level was increased in gastric cancer tissues compared with paired normal adjacent tissues (Fig. 1E, G–I). Furthermore, the expression of PTPN14 in gastric cancer with LNM was elevated compared with gastric cancer tissues without LNM (Fig. 1F, H, I). To evaluate the diagnostic value of PTPN14 in gastric cancer, we analyzed the receiver operating characteristic curve. The expression of PTPN14 could fairly distinguish gastric cancer tissues from normal tissues, with the area under the curve of 0.784 (95% confidence interval = 0.754–0.815, Fig. 1J). However, PTPN14 could hardly distinguish gastric cancer patients with LNM from patients without LNM (Supplemental Fig. 2). Consistent with the online database, the overall survival time of gastric cancer patients with PTPN14 high expression level was shorter than patients with PTPN14 low expression level (Fig. 1K). Our research showed that PTPN14 was an unfavorable prognosis factor in gastric cancer.

We further analyzed the correlation of PTPN14 expression level with clinicopathological parameters (Table 1). The level of PTPN14 expression was irrespective of age, gender, T stage, M stage, Lauren type, mismatch repair (MMR) state, E-cadherin expression, and p53 status. However, PTPN14 expression was positively correlated with the N stage and cTNM stage. In addition, we collected five gastric cancer cell lines and detected PTPN14 expression level by western blot. The protein expression level of PTPN14 was high in HGC-27 and AGS cell lines. In MKN-45, BGC-823, and SGC-7901 cell lines, PTPN14 were lowly expressed (Fig. 1L). We chose AGS, MKN-45, and BGC-823 cell lines for subsequent research.

**Table 1:** PTPN14 expression and correlation with clinicopathological patient characteristics.

Characteristics	PTPN14 expression		Total	*p* Value
	Low	High		
Age				0.6947
≤63	123	98	221	
>63	106	92	198	
Gender				0.0720
Male	163	150	313	
Female	66	40	106	
T stage				0.3952
T1	41	24	65	
T2	23	22	45	
T3	47	35	82	
T4	118	109	227	
N stage				0.0262*
N0	81	48	129	
N1–N3	148	142	290	
M stage				0.5203
M0	223	187	410	
M1	6	3	9	
cTNM stage				0.0445*
I + II	98	63	161	
III + IV	131	127	258	
Lauren				0.3221
Diffused	90	75	165	
Intestinal	79	76	155	
Mixed	60	39	99	
MMR				0.1006
dMMR	22	10	32	
pMMR	207	180	387	
E-cadherin				0.8514
Positive	212	177	389	
Negative	17	13	30	
p53				0.2224
Positive, aberrant	58	56	114	
Negative, aberrant	42	43	85	
Normal	129	91	220	

*p < 0.05, indicates statitical significance.

### PTPN14 is transcriptionally activated by C/EBPβ in gastric cancer

**Fig. 2:**
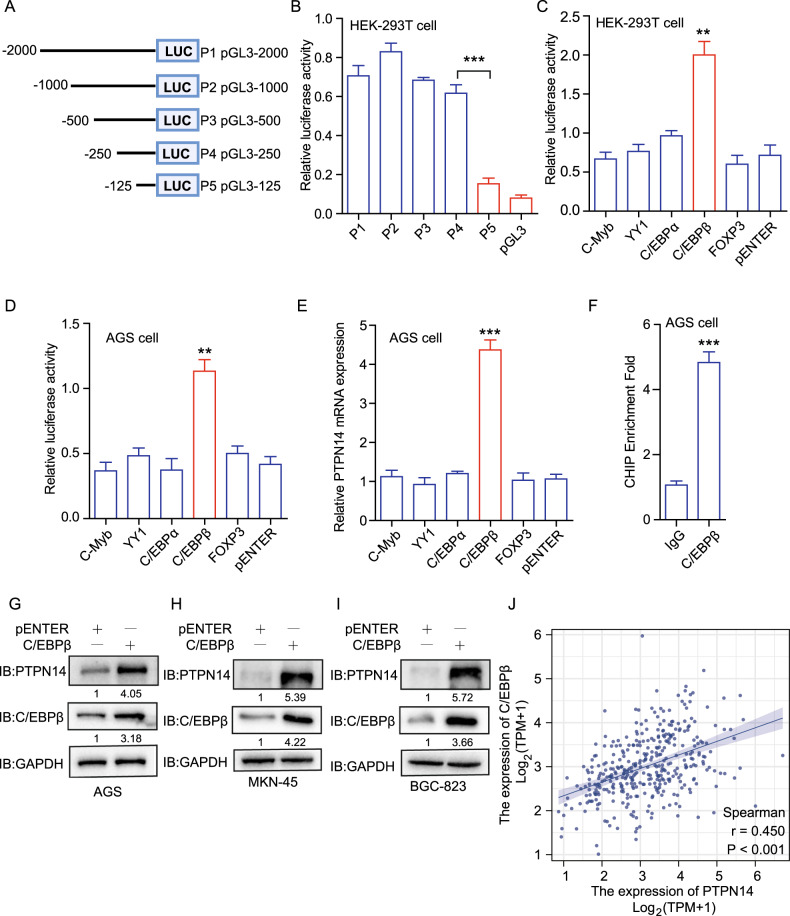
C/EBPβ activates PTPN14 transcription in gastric cancer. **A** Schematic representation of luciferase vectors pGL3-2000(P1), pGL3-1000(P2), pGL3-500(P3), pGL3-250(P4) and pGL3-125(P5). **B** Dual luciferase assay of the PTPN14 promoter fragments in HEK-293T cells indicated that the upstream 125–250 bp was the core region for PTPN14 transcription. ***p < 0.005; cells transfected with P5 were compared with cells overexpressing P4. **C**, **D** Dual luciferase assay showed that CEBP/β increased the transcriptional activity in HEK-293 T cells (**C**) and AGS cells (**D**). **p < 0.01; cells overexpressing CEBP/β were compared with overexpressing pENTER vector. **E** RT-qPCR showed that CEBP/β elevated the mRNA expression level of PTPN14 in AGS cells. ***p < 0.005; cells overexpressing CEBP/β were compared with overexpressing pENTER vector. **F** ChIP-qPCR assays illustrated that the promoter regions precipitated by the anti-CEBP/β antibody were much more enriched than the anti-IgG antibody. ***p < 0.005; promoter regions were precipitated by anti-CEBP/β antibody were compared with anti-IgG antibody. **G**–**I** Western blot showed that CEBP/β promoted PTPN14 expression remarkably in AGS cells (**G**), MKN-45 cells (**H**), and BGC-823 cells (**I**). **J** Correlation analysis of PTPN14 expression and CEBP/β expression in gastric cancer from TCGA database.

In order to explore the underlying mechanism of PTPN14 increased expression level in gastric cancer, we designed a dual luciferase reporter assay. Five regions upstream of the transcription start site (TSS) were subcloned to the pGL3 basic vector (P1 (pGL3-2000), P2 (pGL3-1000), P3 (pGL3-500), P4 (pGL3-250) and P5 (pGL3-125), Fig. 2A). There were no statistical differences in the luciferase activity among P1, P2, P3, and P4. However, the luciferase activity of P5 significantly decreased, indicating that the upstream 125–250 bp region might play key roles in binding with transcription factor (TF; Fig. 2B).

The online websites PROMO and JASPAR predicated that C-Myb, YY1, C/EBPα, C/EBPβ, and FOXP3 were probable TFs binding to the site region from −250 to −125 bp. Then, we overexpressed these TFs separately and detected the luciferase activity. Only C/EBPβ could significantly improve the promoter activities of P1 (Fig. 2C, D). Besides, we detected the mRNA expression level of PTPN14. Only C/EBPβ could upregulate PTPN14 mRNA expression level (Fig. 2E). Meanwhile, overexpression of C/EBPβ significantly upregulated the protein expression level of PTPN14 in AGS cells (Fig. 2G), MKN-45 cells (Fig. 2H), and BGC-823 cells (Fig. 2I). The chromatin immunoprecipitation (ChIP)-PCR assay showed that C/EBPβ could bind with PTPN14 promoter region directly (Fig. 2F). These data showed that C/EBPβ activated PTPN14 transcription in vitro. Furthermore, we analyzed the relationship between C/EBPβ expression level and PTPN14 expression level in gastric cancer from the TCGA database. C/EBPβ expression level had a positive correlation with PTPN14 expression level (*r* = 0.450; Fig. 2J). Taken together, PTPN14 is activated by C/EBPβ at the transcriptional level in gastric cancer.

### PTPN14 promotes gastric cancer cell proliferation, migration, and invasion

**Fig. 3:**
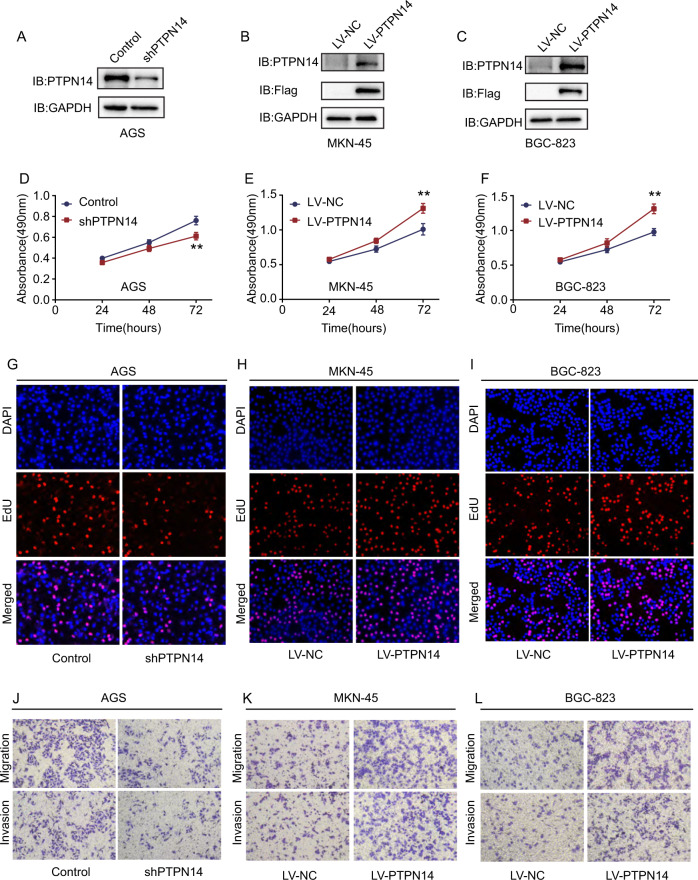
PTPN14 promotes cell proliferation, migration, and invasion in vitro. **A**–**C** Western blot verified the overexpression or knockdown efficacy of PTPN14. In AGS cells, PTPN14 expression was decreased by shRNA (**A**). In BGC-823 cells, LV-PTPN14 stably increased the expression of PTPN14 (**B**). In MKN-45 cells, LV-PTPN14 stably increased the expression of PTPN14 (**C**). **D**–**F** CCK-8 assays showed that PTPN14 knockdown inhibited cell proliferation in AGS cells (**D**), while overexpression of PTPN14 promoted cell proliferation in MKN-45 cells (**E**) and BGC-823 cells (**F**). **p < 0.01; cells overexpressing PTPN14 or knockdown PTPN14 were compared with control cells. **G**–**I** EDU assays showed that PTPN14 promoted cell proliferation. In AGS cells, the shPTPN14 group showed fewer EDU-positive cells than the control group (**G**). The LV-PTPN14 group showed more EDU-positive cells than the control group in MKN-45 cells (**H**) and BGC-823 cells (**I**). **J**–**L** Transwell assays indicated that PTPN14 promoted gastric cancer cell migration and invasion. In AGS cells, the shPTPN14 group showed fewer migrating or invading cells than the control group (**J**). The LV-PTPN14 group showed more migrating or invading cells than the control group in MKN-45 cells (**K**) and BGC-823 cells (**L**).

To explore the function of PTPN14 in gastric cancer, we established and identified three stable cell lines (Fig. 3A–C). We transformed AGS cells with lentivirus (shPTPN14) to knockdown PTPN14 expression and named it the shPTPN14 group. We transfected MKN-45 and BGC-823 cell lines with lentivirus (LV-PTPN14) stably to increase PTPN14 expression and named it the LV-PTPN14 group. CCK-8 and EDU assays showed that PTPN14 overexpression could promote gastric cancer cell proliferation and PTPN14 knockdown could inhibit gastric cancer cell proliferation (Fig. 3D–I and Supplemental Fig. 3A–C). The transwell assay indicated that the knockdown of PTPN14 could suppress gastric cancer cell migration and invasion in AGS cells (Fig. 3J and Supplemental Fig. 4A). Meanwhile, overexpressing PTPN14 could promote gastric cancer cell migration and invasion in MKN-45 and BGC-823 cells (Fig. 3K, L and Supplemental Fig. 4B, C). Therefore, PTPN14 could promote gastric cancer cell proliferation, migration, and invasion in vitro.

### The FERM domain of PTPN14 plays a major role in promoting gastric cancer progression

**Fig. 4:**
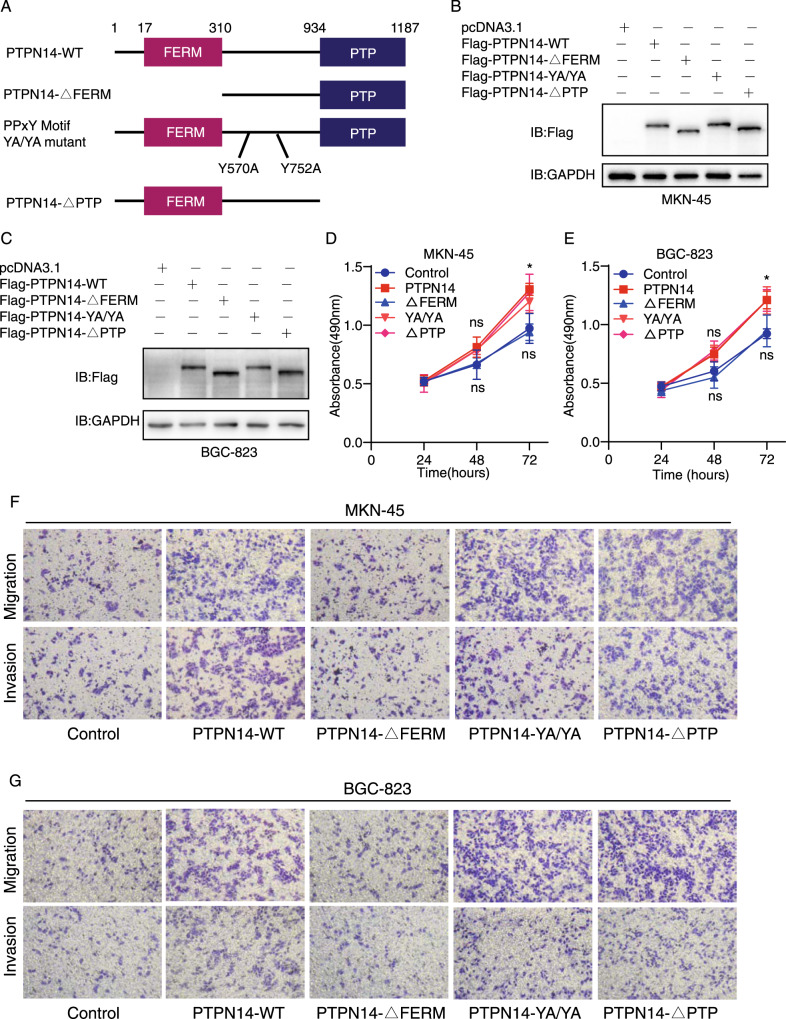
PTPN14 promotes gastric cancer cell proliferation, migration, and invasion mainly through its FERM domain. **A** Schematic representation of PTPN14 wild-type plasmid and its truncated plasmids. **B**, **C** Western blot verified the expression of PTPN14 wild-type plasmid and PTPN14 truncated plasmids in MKN-45 cells (**B**) and BGC-823 cells (**C**). **D**, **E** CCK-8 assays indicated that PTPN14-ΔFERM diminished its ability to promote cell proliferation in MKN-45 cells (**D**) and BGC-823 cells (**E**). *p < 0.05; cells overexpressing PTPN14 wild type plasmids, YA/YA mutant plasmids or ΔPTP mutant plasmids were compared with control cells. **F**, **G** Transwell assays showed that PTPN14-ΔFERM could not prompt cell migration and invasion in MKN-45 cells (**F**) and BGC-823 cells (**G**).

PTPN14 is a member of the classical protein tyrosine phosphatase subfamily, which is mainly organized into three parts: the FERM domain, the PTP domain, and the middle part [8]. In the middle part, there are two PPxY motifs playing important roles in protein–protein interactions. To make sure the key domain of PTPN14 in promoting gastric cancer progression, we subcloned three PTPN14 mutant plasmids: PTPN14-ΔFERM (deletion of the FERM domain, 17–310 amino acids), PTPN14-YA/YA mutant (the PPxY motif mutant, Y570A/Y752A), and PTPN14-ΔPTP (deletion the PTP domain, 934–1187 amino acids) (Fig. 4A). Then we transformed MKN-45 cells and BGC-823 cells with PTPN14 wild type plasmids and these mutant plasmids separately. The transfection efficiency was verified by western blot assay (Fig. 4B, C). Furthermore, we transformed MKN-45 cells or BGC-823 cells with GFP-PTPN14 plasmid under the same condition to indicate the transfection efficacy. The transfection efficacy was almost 90% (Supplemental Fig. 5). CCK-8 assays showed that PTPN14, PTPN14-YA/YA, and PTPN14-ΔPTP could enhance cell proliferation significantly. However, PTPN14-ΔFERM could hardly promote cell proliferation, indicating that the FERM domain played a vital role in promoting cell proliferation (Fig. 4D, E). Transwell assays showed that only the PTPN14-ΔFERM mutant lost the capacity of promoting cell migration and invasion (Fig. 4F, G and Supplemental Fig. 6A, B). In summary, the FERM domain of PTPN14 is the key domain in promoting gastric cancer cell proliferation, migration, and invasion.

### PTPN14 positively regulates PI3KA/AKT/mTOR pathway in gastric cancer

**Fig. 5:**
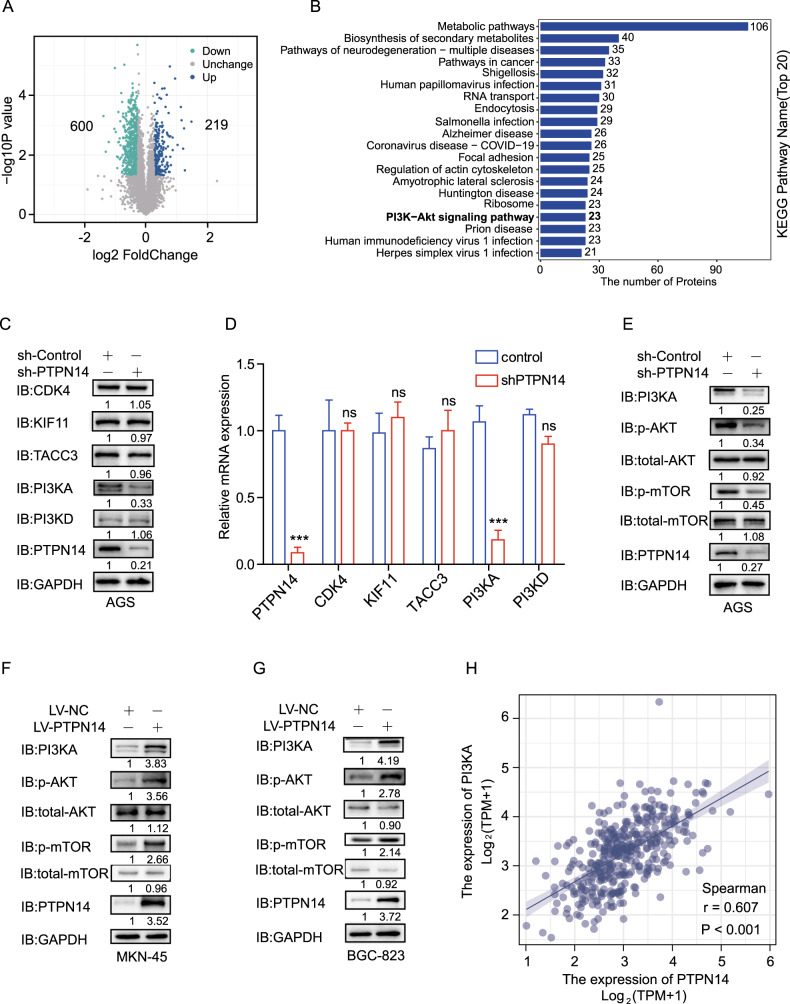
The PI3KA/AKT/mTOR pathway was elevated by PTPN14 in gastric cancer. **A** The comparative proteomic analysis showed the number of proteins with significant alterations in AGS-shPTPN14 cells. **B** The top 20 KEGG annotations of the comparative proteomic analysis included the PI3K/AKT pathway (bold highlight). **C** Western blot validation of the selected five key proteins showed that the PI3KA expression was decreased by PTPN14 knockdown. **D** RT-qPCR indicated that the mRNA expression of PI3KA was downregulated by PTPN14 knockdown. ***p < 0.005; the mRNA expression level of PTPN14 or PI3KA of shPTPN14 group was compared with the control group. **E**–**G** Western blot showed that PTPN14 regulated the PI3KA/AKT/mTOR pathway. In AGS shPTPN14 group, the expression level of PI3KA and phosphorylation levels of AKT and mTOR were obviously decreased (**E**). The expression level of PI3KA and phosphorylation levels of AKT and mTOR were obviously increased in the LV-PTPN14 group in MKN-45 cells (**F**) and BGC-823 cells (**G**). **H** Correlation analysis of PTPN14 expression and PI3KA expression in gastric cancer from TCGA database.

In order to pursue the underlying molecular mechanism of PTPN14 enhancing gastric cancer progression, we performed a comparative proteomic analysis. In the AGS shPTPN14 group, there were 600 upregulated proteins and 219 downregulated proteins (Fig. 5A). Furthermore, the KEGG pathway annotation on the significantly differentially expressed proteins indicated that the PI3K/AKT pathway may be changed dramatically (Fig. 5B). It is well known that PI3KA is the initiating molecule of PI3KA/AKT/mTOR pathway [22]. We chose and verified 5 key proteins. In AGS cells, the changes in protein levels of CDK4, KIF11, TACC3, and PI3KD in the control group and shPTPN14 group were not obvious. However, the PI3KA expression was significantly downregulated in the shPTPN14 group (Fig. 5C). Consistently with the changes in protein expression, the PI3KA mRNA expression was much lower in the shPTPN14 group than in the Control group in AGS cells. The mRNA expression of CDK4, KIF11, TACC3, and PI3KD had no evident difference in the two groups (Fig. 5D). In addition, we verified the PI3KA/AKT/mTOR pathway by western blot in three different gastric cancer cell lines. In AGS cells, when PTPN14 expression was significantly inhibited by lentivirus, the expression level of PI3KA and phosphorylation level of AKT and mTOR were downregulated obviously (Fig. 5E). In MKN-45 and BGC-823 cells, the PI3KA/AKT/mTOR pathway was prompted significantly by the elevation of PTPN14 expression (Fig. 5F, G). To illustrate the positive regulation of PTPN14 on PI3KA in vivo, we analyzed the correlation between PTPN14 expression level and PI3KA expression level in gastric cancer from the TCGA database. There was a significant and positive correlation between the PTPN14 expression level and PI3KA expression level (*r* = 0.607; Fig. 5H). In summary, PTPN14 promotes the PI3KA/AKT/mTOR pathway in gastric cancer.

### PTPN14 promotes PI3KA/AKT pathway by binding and promoting NFkB nucleus translocation

**Fig. 6:**
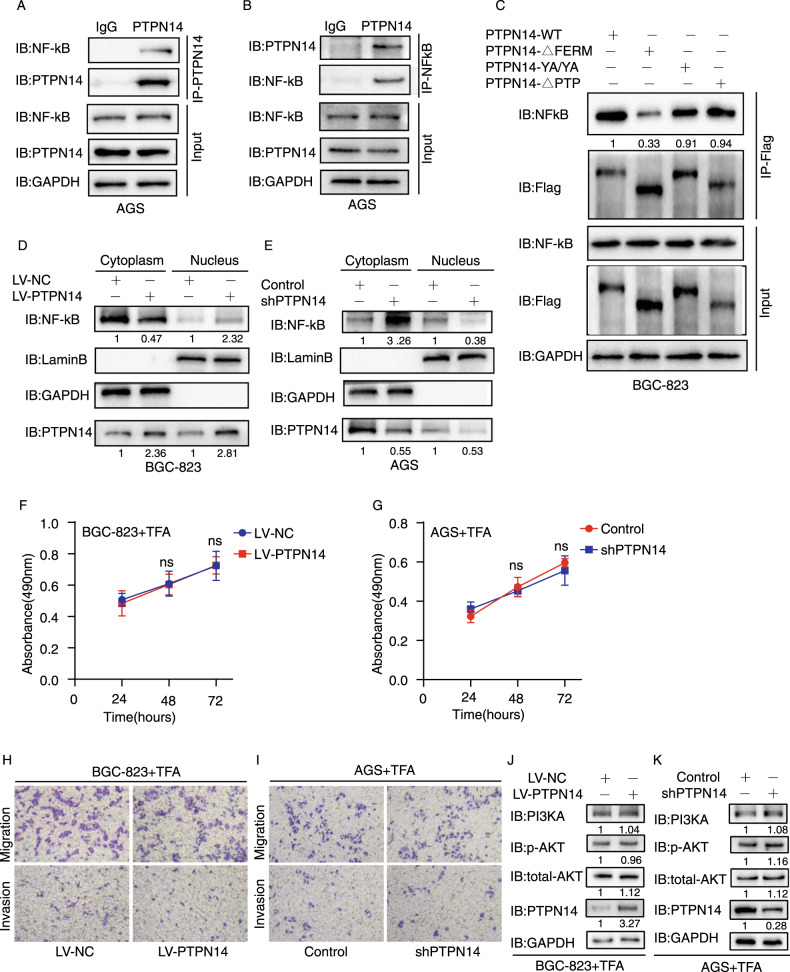
PTPN14 regulates the PI3KA/AKT pathway through NFkB nucleus translocation. **A**, **B** The CO-IP results showed that PTPN14 interacted with NFkB. **C** The CO-IP assays by PTPN14 truncated plasmids showed that the amount of NFkB binding with PTPN14-ΔFERM was much lower than PTPN14 wild type. **D**, **E** The nucleus and cytoplasm separation assays showed that the amount of NFkB in the nucleus was increased in BGC-823 cells by PTPN14 overexpressing (**D**) and the amount of NFkB in the nucleus was decreased in AGS cells by PTPN14 reduction (**E**). **F**, **G** After incubating with PTD-p65-P1 Peptide TFA in BGC-823 cells (**F**) and AGS cells (**G**), alteration of PTPN14 expression did not influence cell proliferation. **H**, **I** In BGC-823 cells (**H**) and AGS cells (**I**) incubating with PTD-p65-P1 Peptide TFA, the migrating or invading cell numbers were not altered by the PTPN14 expression level. **J**, **K** After incubating with PTD-p65-P1 Peptide TFA in BGC-823 cells (**J**) and AGS cells (**K**), alteration of PTPN14 expression did not influence the PI3KA/AKT pathway.

It was natural to explore the detailed mechanism of PTPN14 upregulating PI3KA expression subsequently. Previous research reported that PTPN14 could bind with YAP and promote SMAD3 nucleus translocation [16]. Also, PTPN14 could translocate into the nucleus [15]. These researches suggested that PTPN14 may regulate TFs translocating into the nucleus. After searching related works of literature, we found that NFkB, as a TF could regulate PI3KA expression [23]. Thus, we hypothesized that PTPN14 could bind with NFkB and promote NFkB nucleus translocation to upregulate PI3KA transcription. To check our hypothesis, we first performed a Co-IP assay to detect whether PTPN14 could bind with NFkB. When immunoprecipitation with anti-PTPN14 antibody, NFkB could be detected by Western blot (Fig. 6A). Conversely, when immunoprecipitation with anti- NFkB antibody, PTPN14 also could be detected (Fig. 6B). In addition, to clarify the binding region of PTPN14 and NFkB, we performed CO-IP assay using truncated or mutated PTPN14 plasmids. The amount of NFkB binding with PTPN14-ΔFERM was much lower than with PTPN14 wild type. However, the deletion of the PTP domain or PPxY motif mutation did not affect the combination of PTPN14 and NFkB (Fig. 6C). These results indicated that PTPN14 combined with NFkB mainly through the FERM domain.

NFkB, as a TF could move between the nucleus and cytoplasm. Previous research showed that the translocation of NFkB was regulated by cytokines or binding proteins [24]. To explore whether PTPN14 affected NFkB nucleus translocation, we carried out a nucleus cytoplasm separation experiment. GAPDH is the marker protein of cytoplasm, and Lamin B is the nucleus marker protein. To confirm the nucleus cytoplasm separation efficacy, we tested the expression of GADPH and NFkB in the cytoplasm and nucleus separately, confirming qualified separation (Fig. 6D, E). When PTPN14 was overexpressed in BGC-823 cells, the expression of PTPN14 both in the cytoplasm and nucleus was increased. Also, the NFkB expression level in the nucleus was increased with the increased expression of PTPN14 (Fig. 6D). However, the NFkB expression level in the cytoplasm was lower in the LV-PTPN14 group than the LV-NC group (Fig. 6D). In accordance with this, when the expression of PTPN14 was suppressed in AGS cells, the NFkB expression in the nucleus was decreased and the NFkB expression in the cytoplasm was increased (Fig. 6E). Further experiments verified that PTPN14 did not affect the expression of NFkB (Supplemental Fig. 7A, B). Thus, PTPN14 interacting with NFkB may promote NFkB nuclear translocation to increase the transcription capacity.

**Fig. 7:**
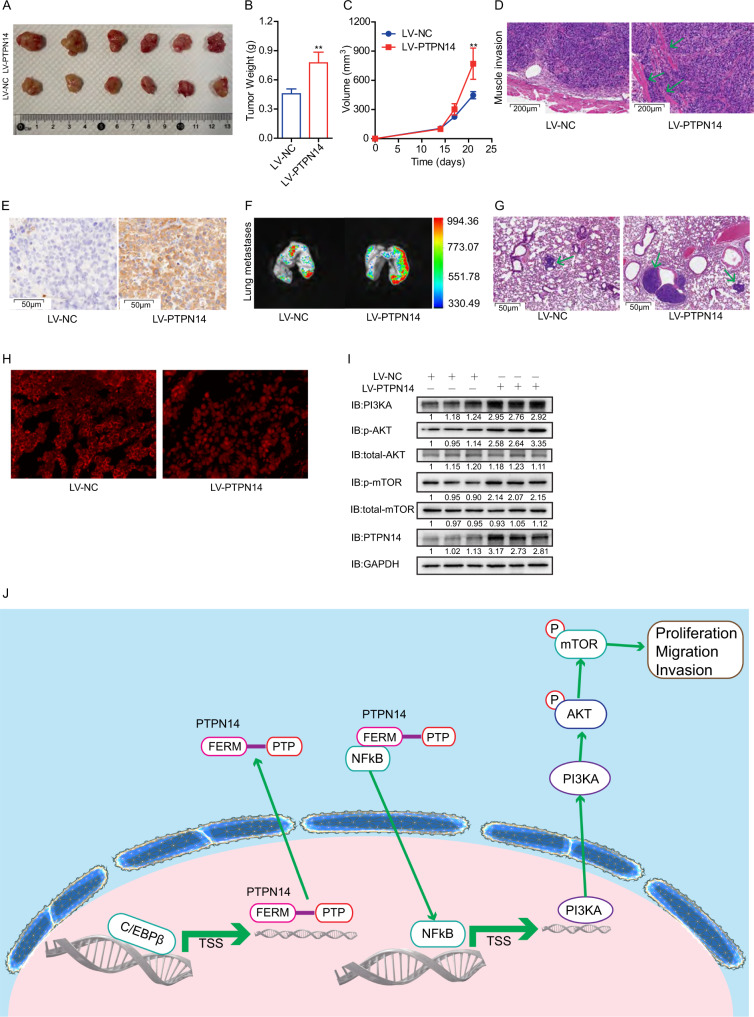
PTPN14 promotes gastric cancer cell proliferation, migration, and invasion by PI3KA/AKT/mTOR pathway through NFkB in mice models. **A** The presentation of xenograft tumors from nude mice implanted with BGC-823 cells stably transfected with LV-PTPN14 or LV-NC. **B** The average tumor weight of the xenograft tumors. **p < 0.01; xenograft tumors of LV-PTPN14 group were compared with LV-NC group. **C** Tumor growth curves showed that PTPN14 overexpression obviously accelerated tumor growth in vivo. **D** The hematoxylin–eosin (HE) staining of xenograft tumors showed that the LV-PTPN14 group displayed more muscle invasion (the green arrow), however, the LV-NC group was well encapsulated. **E** The IHC staining of PTPN14 verified the PTPN14 expression in xenograft tumors from the LV-PTPN14 group and LV-NC group. **F** The tumors from the lungs of the pulmonary metastasis model were imaged. **G** The HE staining of lungs from the pulmonary metastasis model showed that the LV-PTPN14 group developed more and larger tumors (the green arrow). **H** IF of NFkB showed that the NFkB is mainly expressed in the cytoplasm in the LV-NC group; however, NFkB is expressed both in the nucleus and cytoplasm in the LV-PTPN14 group. **I** Western blot of xenograft tumors illustrated that PTPN14 promoted the PI3KA/AKT/mTOR pathway in gastric cancer. **J** Schematic illustration of the mechanisms of PTPN14 in gastric cancer. The CEBP/β activates PTPN14 expression. The elevated PTPN14 binds with NFkB through its FERM domain and prompts NFkB nucleus translocation. The nucleus NFkB transcriptionally activates PI3KA expression and prompts PI3KA/AKT/mTOR pathway to promote gastric cancer cell proliferation, migration, and invasion.

PTD-p65-P1 Peptide TFA is a selective inhibitor of NFkB, which suppresses the nucleus translocation of NFkB [21]. We incubated BGC-823 cells or AGS cells with TFA and then detected the efficacy of PTPN14 on cell proliferation, migration, and invasion. We found that TFA could eliminate the promoting effect of PTPN14 on gastric cancer proliferation, migration, and invasion (Fig. 7F–I and Supplemental Fig. 8). In the meantime, the promoting effect of PTPN14 on the PI3K/AKT pathway was blocked by TFA (Fig. 7J, K). Therefore, NFkB nucleus translocation was vital and necessary for PTPN14 to exert gastric cancer’s supporting role.

### PTPN14 promotes gastric cancer cell proliferation, migration, and invasion in mice model

As the last step, we used mice models to verify the above findings. We injected BGC-823 cells stably transfected with LV-PTPN14 or LV-NC subcutaneously into nude mice. The xenograft tumor growth was faster in the LV-PTPN14 group than those in the LV-NC group. Consequently, the xenograft tumor weight and volume were significantly larger in the LV-PTPN14 group than those in the LV-NC group (Fig. 7A–C). Besides, the tumors in the LV-PTPN14 group showed local invasion into surrounding muscle tissues, but the tumors in the LV-NC group were well-encapsulated (Fig. 7D). Finally, we confirmed the PTPN14 expression level in xenograft tumors by IHC. The expression level of PTPN14 was much higher in the LV-PTPN14 group than in the LV-NC group (Fig. 7E). Furthermore, we established a pulmonary metastasis model by tail vein injection of stable transfected BGC-823 cells to the nude mice. The pulmonary imaging and hematoxylin and eosin (HE) staining indicated that more and larger pulmonary metastatic tumors developed in the LV-PTPN14 group than in the LV-NC group (Fig. 7F, G). To sum up, PTPN14 could enhance gastric cancer cell proliferation and metastasis in vivo.

Moreover, we tested NFkB subcellular location in the xenograft tumors by immunofluorescence. In the LV-NC group, NFkB was found mainly in the cytoplasm. However, in the LV-PTPN14 group, NFkB is located both in the cytoplasm and nucleus (Fig. 7H). These results verified that PTPN14 could promote NFkB nucleus translocation. We tested the PI3K/AKT/mTOR pathway in the xenograft tumors. In line with the above results, the protein expression level of PI3KA and phosphorylation levels of AKT and mTOR were much higher in the LV-PTPN14 group than in the LV-NC group (Fig. 7I).

In conclusion, the CEBP/β transcriptionally activates PTPN14 and then PTPN14 binds with NFkB through the FERM domain and promotes NFkB nucleus translocation. Furthermore, NFkB upregulates the expression of PI3KA and activates PI3KA/AKT/mTOR pathway to promote gastric cancer cell proliferation, migration, and invasion (Fig. 7J).

## Discussion

PTPN14 is a FERM domain-containing protein, which belongs to the protein tyrosine phosphatase subfamily. PTPN14 is constituted by the FERM domain, PTP domain, and the middle segment [8, 9]. There was a little report on the middle segment, except for the PPxY motif. The PPxY motif and the FERM domain of PTPN14 mainly function by interacting with other proteins. The PTP domain of PTPN14 has poor phosphatase activity because of Ile939 in the pY-loop [9]. However, PTPN14 exerts the role of a tumor suppressor gene by dephosphorylating the specific substrate protein [11]. There are many studies of PTPN14 in cancer, indicating that PTPN14 plays a vital role in cancer. For example, PTPN14 negatively regulates the YAP pathway in cervical cancer and pancreatic cancer [25, 26]. Besides, PTPN14 is very important in the immune response. Previous research indicated that PTPN14 promoted the TGFβ signaling pathway in rheumatoid synoviocytes by interaction with YAP [16]. In acute liver failure, PTPN14 aggravates the NFkB signaling pathway by promoting SOCS7 degradation [17]. However, the roles and underlying molecular mechanisms of PTPN14 in gastric cancer are poorly understood.

In the present research, we collected gastric cancer samples from the Second Hospital of Shandong University. By IHC staining and scoring, we found that PTPN14 was upregulated in gastric cancer, especially with LNM. The expression level of PTPN14 could help pathologists to distinguish gastric cancer tissues from normal gastric tissues, indicating the diagnostic value of PTPN14 in gastric cancer. To see the mechanism of PTPN14 upregulation, we clarified the promoter region and pointed out CEBPβ as an effective TF. By overexpression or knockdown of PTPN14, we demonstrated that PTPN14 could promote gastric cancer proliferation, migration, and invasion. Furthermore, the FERM domain of PTPN14 was the key domain in gastric cancer promotion.

The comparative proteomic analysis combined with western blot verification indicated that PI3KA expression was regulated by PTPN14. PI3KA is the initial molecule in the PI3K/AKT/mTOR pathway. In gastric cancer, PTPN14 promoted the PI3K/AKT/mTOR pathway. The PI3K/AKT/mTOR pathway is crucial to cancer cell proliferation, migration, and invasion. Taken together, PTPN14 promotes gastric cancer cell proliferation, migration, and invasion by PI3K/AKT/mTOR pathway. Thus, the key point was the definite mechanism of PTPN14 upregulating PI3KA expression. Previous research indicated that NFkB could activate PI3KA expression. NFkB is a TF, which can move between the nucleus and cytoplasm [24]. The current report showed that PTPN14 could interact with YAP, a TF, and promoted the nucleus localization of SMAD3 [16]. Besides, previous research indicated that PTPN14 was a phosphatase, which can translocate from the cytoplasm to the nucleus [15]. Based on the above literature research, we hypothesized that PTPN14 interacted with NFkB and promoted NFkB nuclear translocation to initiate PI3KA expression, thus activating the PI3K/AKT/mTOR pathway. Our results showed that PTPN14 could bind with NFkB through the FERM domain. The expression of PTPN14 promoted the nucleus localization of NFkB. To verify the necessity of NFkB in PTPN14 upregulating PI3KA expression, we introduced the NFkB inhibitor. In our research, the NFkB inhibitor could diminish the gastric cancer-prompting effect of PTPN14. Finally, we verified these findings in mice models. Our research illustrated the vital roles of PTPN14 in gastric cancer.

## Conclusions

In conclusion, we first demonstrated the function and the underlying molecular mechanism of PTPN14 in gastric cancer. PTPN14 was upregulated by CEBPβ in gastric cancer. High expression of PTPN14 is associated with the cTNM stage, N stage, and poor prognosis in gastric cancer. Furthermore, PTPN14 combined with NFkB through the FERM domain and promoted NFkB nucleus translocation. Thus, NFkB activated PI3KA expression and prompted PI3KA/AKT/mTOR pathway, accelerating gastric cancer cell proliferation, migration, and invasion.

## Supplementary information

Supplemental Figure1-8 and Supplemental Table 1

Reproducibility checklist

## Data Availability

Data supporting the conclusions of this research are shown in this article and the Supplemental Files.
