# Supplementary material for: PTPN14 promotes gastric cancer progression by PI3KA/AKT/mTOR pathway
Source: Cell Death Dis. 2023 Mar 10;14(3):188. doi: 10.1038/s41419-023-05712-4 (PMC10006225; doi:10.1038/s41419-023-05712-4)

Supplementary Material for:

**PTPN14 Promotes Gastric Cancer Progression by PI3KA/AKT/mTOR Pathway**

**This supplementary material includes 1 table and 7figures.**

**Supplemental table 1.** Primer sequence used for RT-QPCR.

| Name | Primers Sequence |
| --- | --- |
| PTPN14-Forward | CGACTTCTGGCAGATGGTGT |
| PTPN14-Reverse | GTGGCTTTTGGTTCGTCCAC |
| PI3KA-Forward | TTGGAAGCAGCAACCGAAAC |
| PI3KA-Reverse | CTTTACTTCGCCGTCCACCA |
| CDK4-Forward | GGTGACAAGTGGTGGAACAG |
| CDK4-Reverse | GCCCATTCAGGTCAAAGATT |
| KIF11-Forward | TACGACACCACAGAGGAA |
| KIF11-Reverse | CCACACCAGCATCTACAG |
| TACC3-Forward | CCTCTTCAAGCGTTTTGAGAAAC |
| TACC3-Reverse | GCCCTCCTGGGTGATCCTT |
| PI3KD-Forward | CATATGTGCTGGGCATTGGC |
| PI3KD-Reverse | TTTCACAGTAGCCCCGGAAC |
| CHIP-Forward | CAAGTAGCTGGGACTACAGGC |
| CHIP-Reverse | AAGCATGGTGGCTCATGTGT |

**Supplemental figure 1.** Presentation of the gastric tissue microarray. (A) The pattern diagram of gastric cancer tissues and the paired normal gastric tissues. (B) The thumbnail view of the HE staining slides and IHC staining of PTPN14.


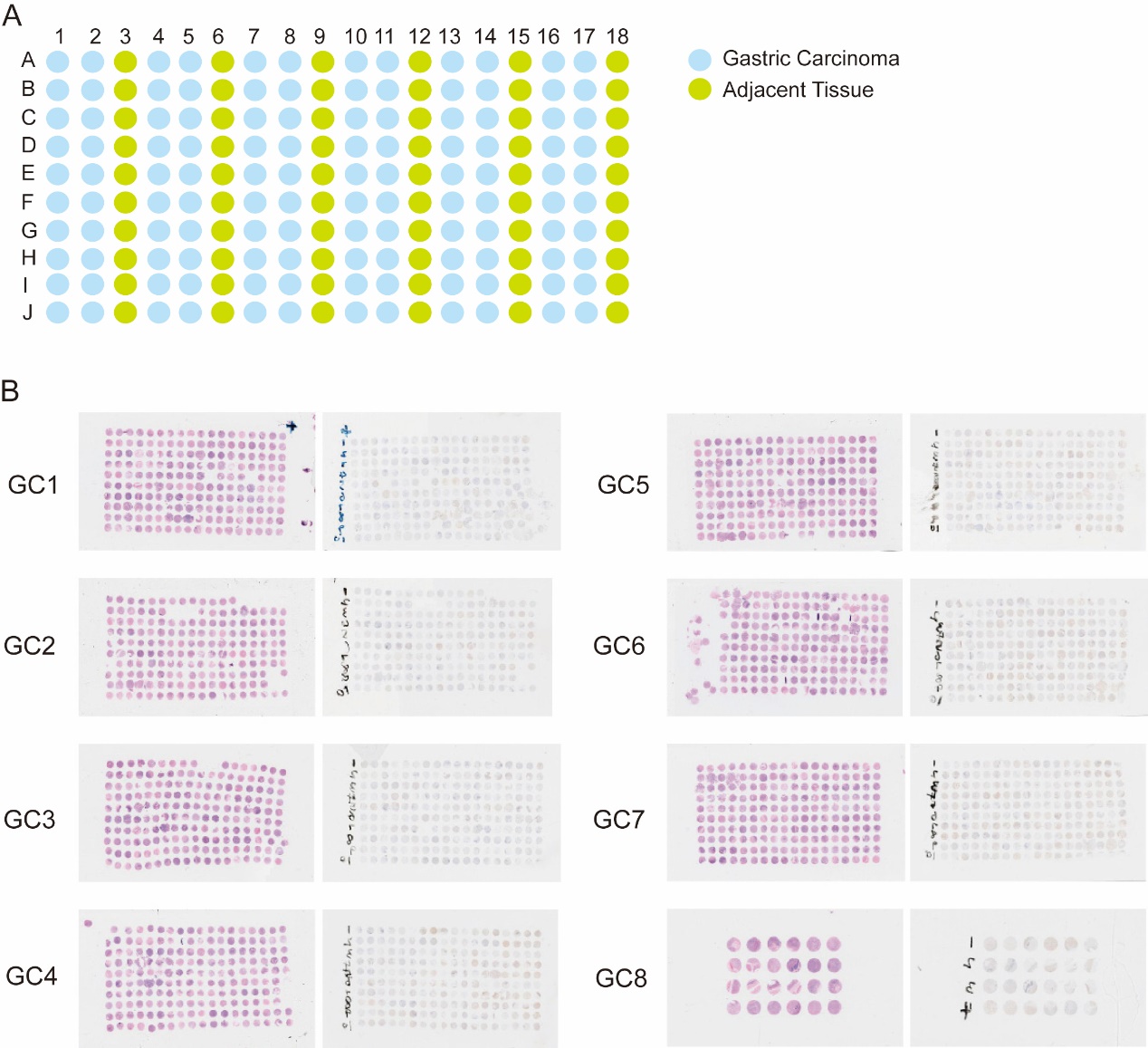


**Supplemental figure 2.** The ROC curve showed that the expression of PTPN14 could hardly distinguish gastric cancer tissues with LNM from gastric cancer tissues without LNM.


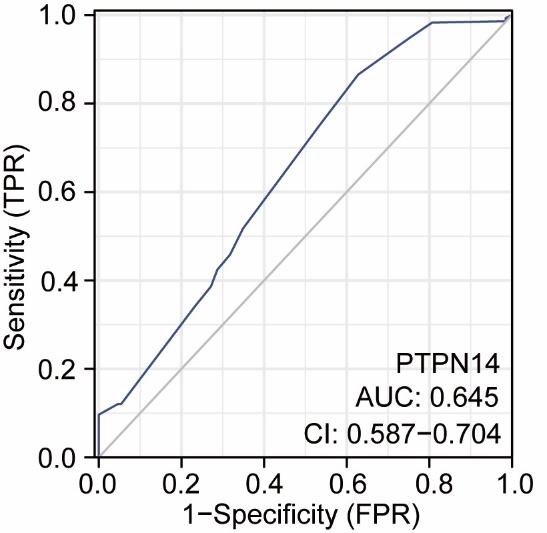


**Supplemental figure 3.** Bar graph representation of proportion of EDU positive cells in AGS cells (A), MKN-45 cells (B) and BGC-823 cells (C) (related to Fig. 3G-3I).


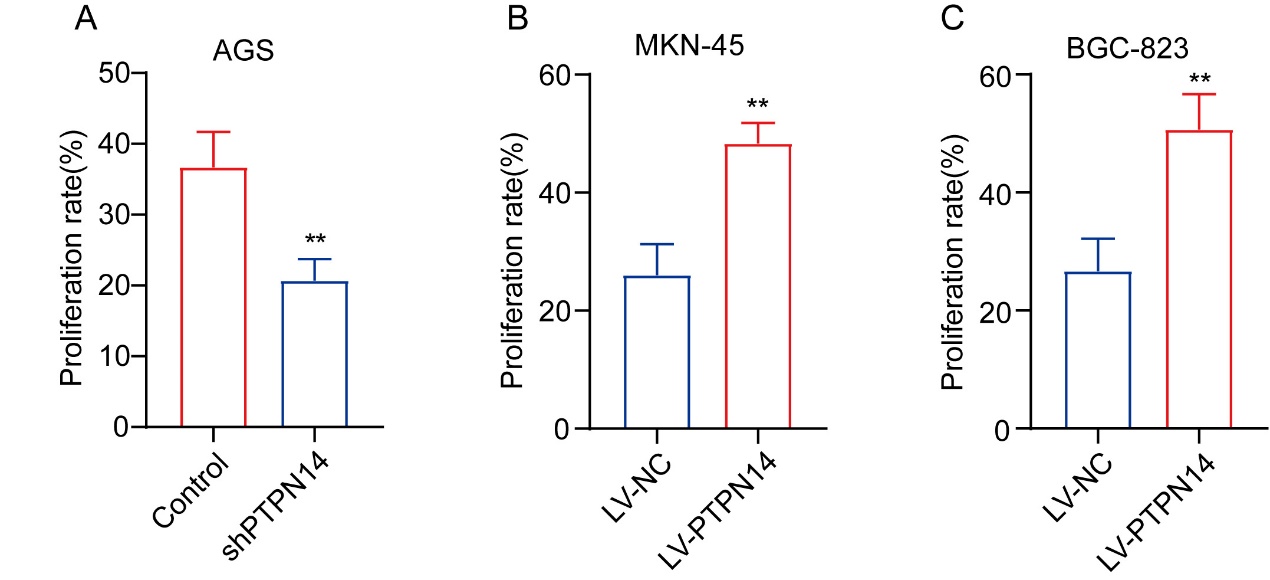


**Supplemental figure 4.** Bar graph representation of migration and invasion cell numbers in AGS cells (A), MKN-45 cells (B) and BGC-823 cells (C) (related to Fig. 3J-3L).


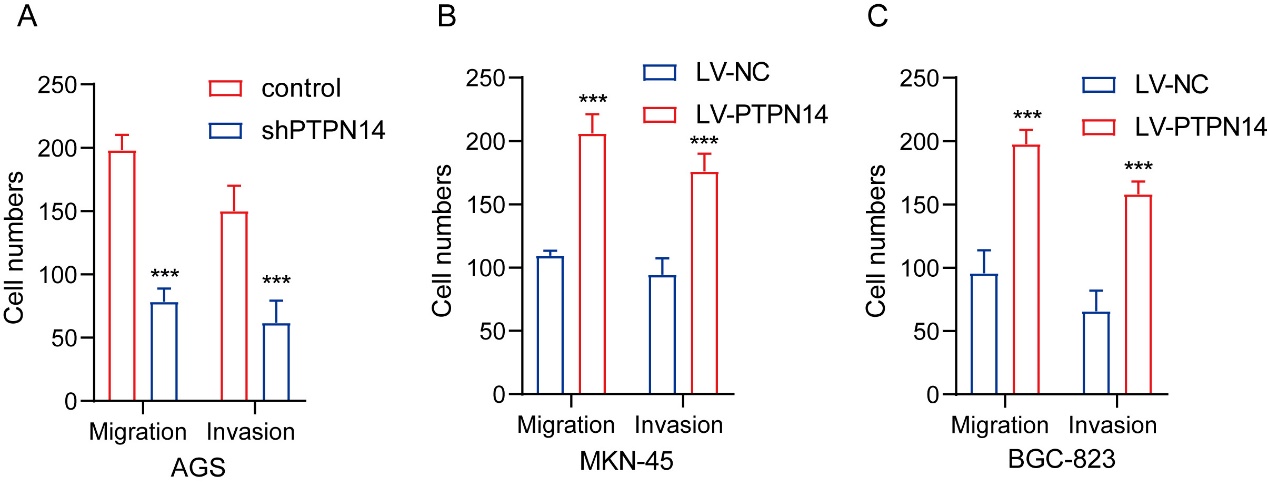


**Supplemental figure 5.** The transfection efficacy of GFP-PTPN14 in MKN-45 cells (A) and BGC-823 cells (B) was almost 90%.


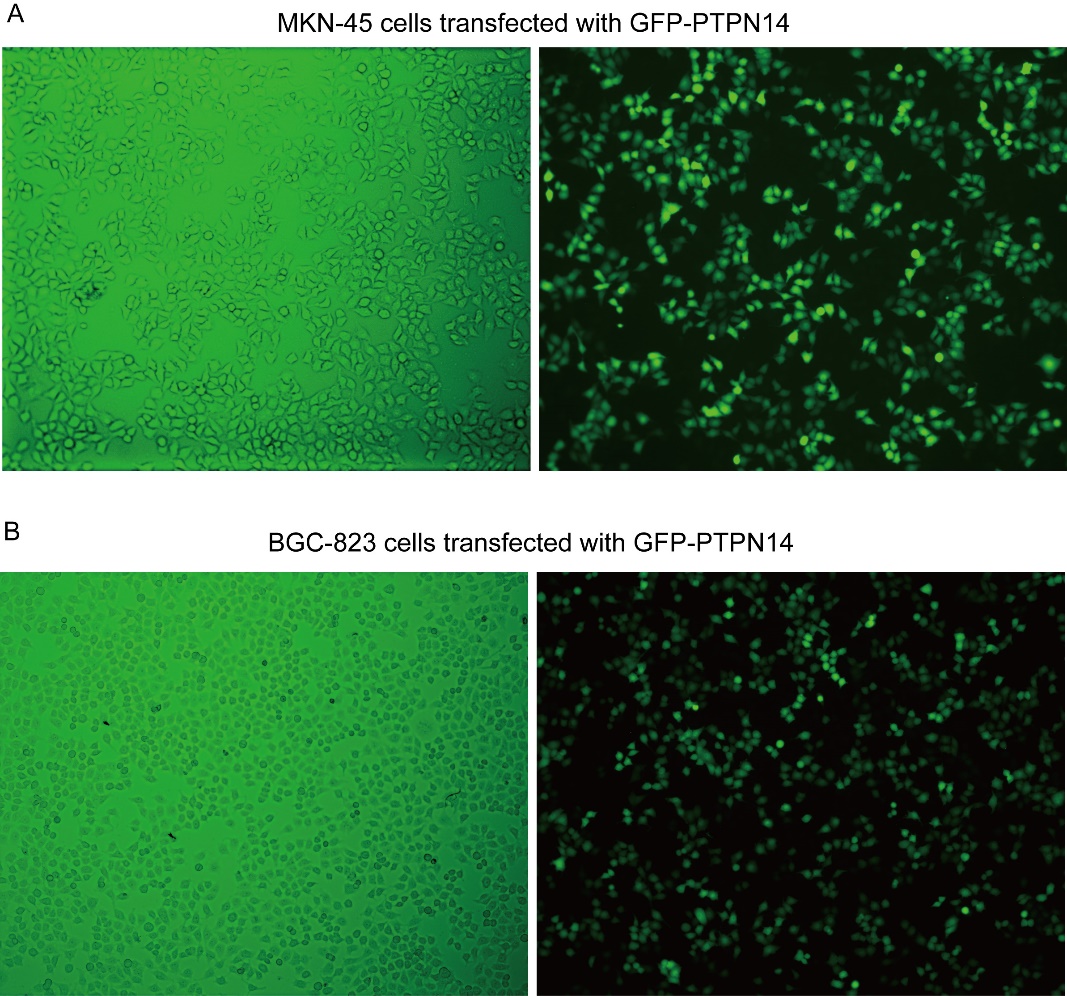


**Supplemental figure 6.** Bar graph representation of migration and invasion cell numbers in MKN-45 cells (A) and BGC-823 cells (B) (related to Fig. 4F-4G).


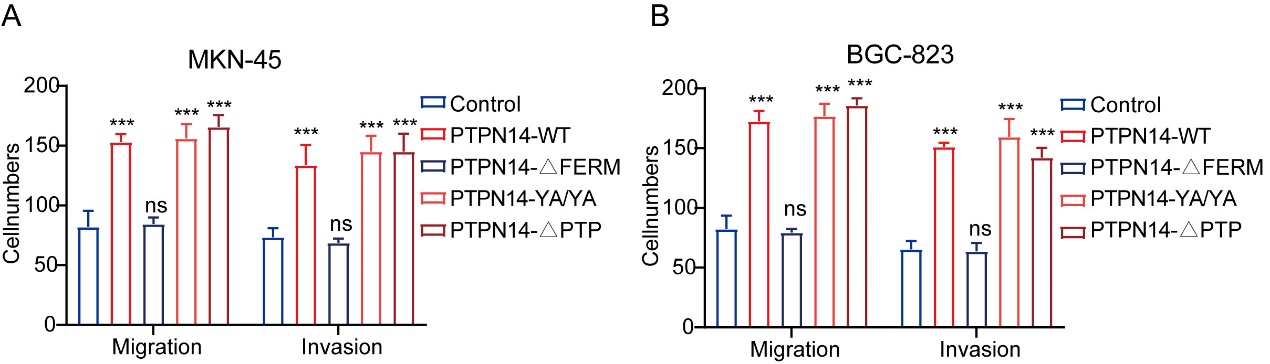


**Supplemental figure 7.** PTPN14 did not affect the expression of NFkB. (A) In BGC-823 cells, PTPN14 overexpression did not affect the expression level of NFkB. (B) In AGS cells, PTPN14 knockdown did not affect the expression level of NFkB.


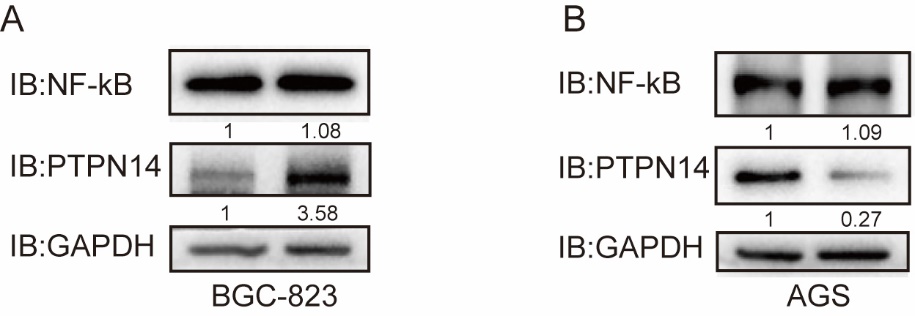


**Supplemental figure 8.** Bar graph representation of migration and invasion cell numbers in BGC-823 cells (A) and AGS cells (B) (related to Fig. 6H-6I).


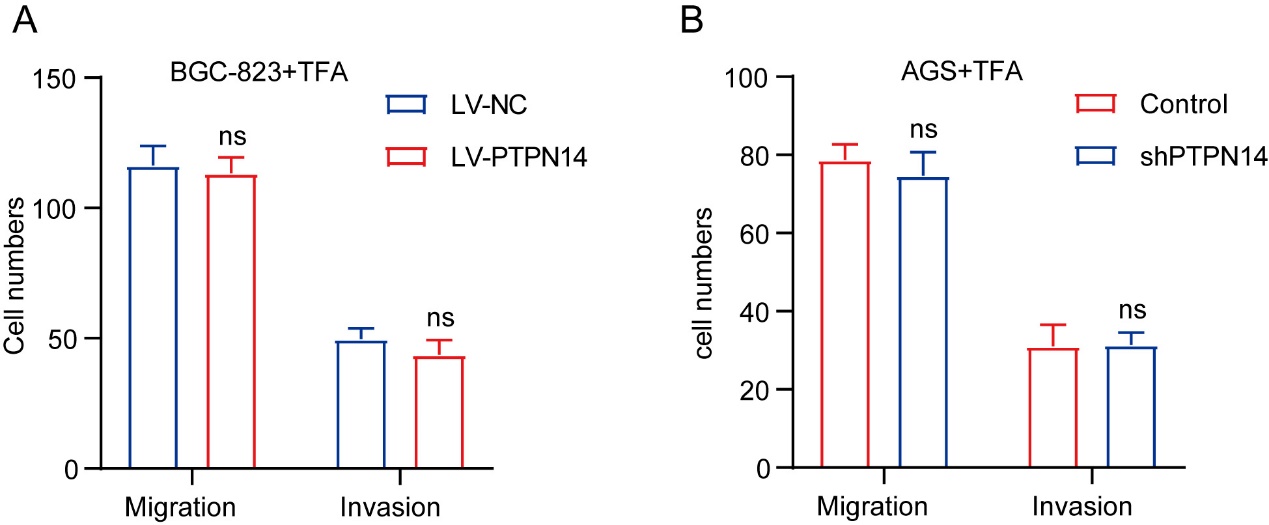


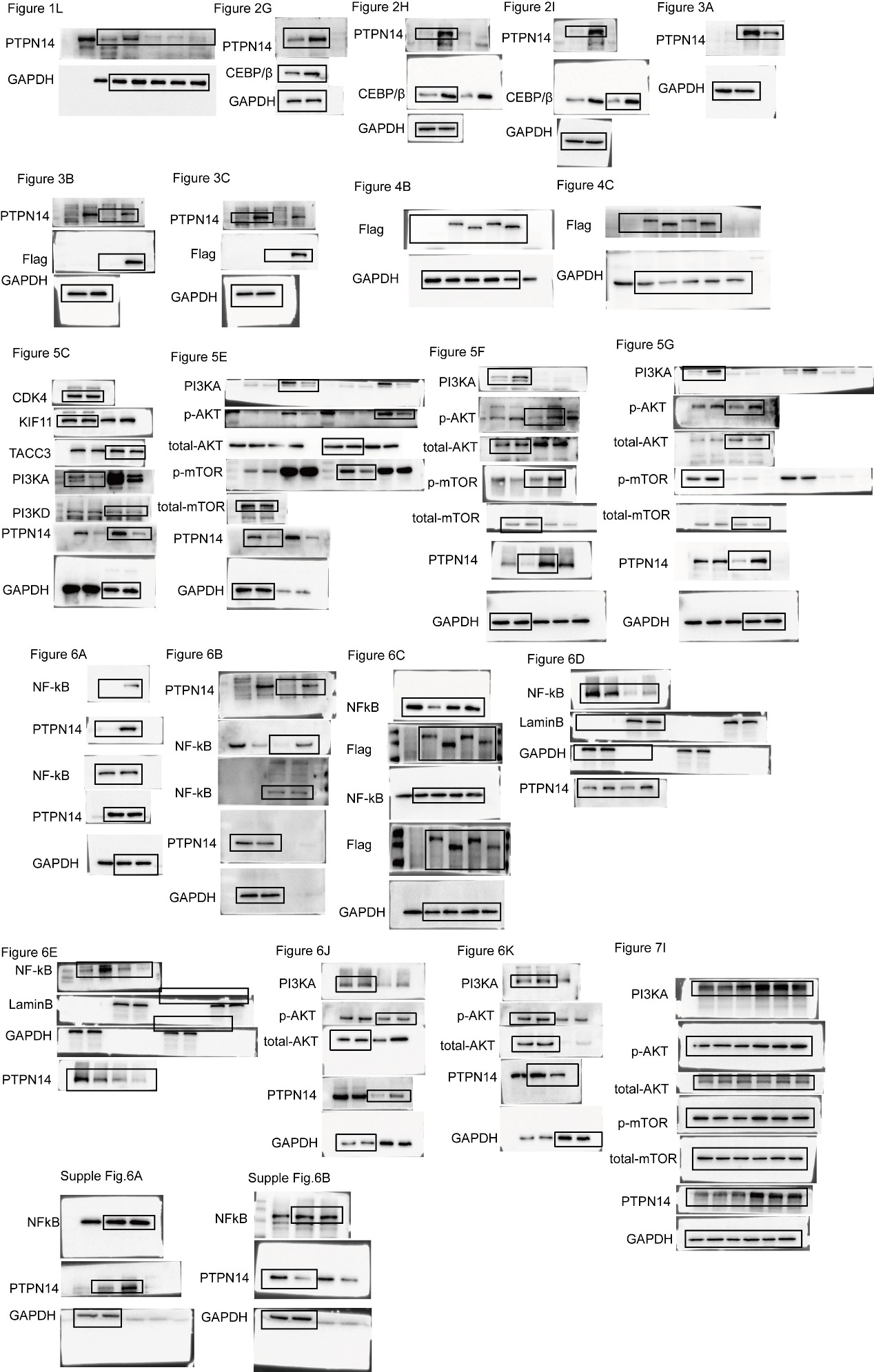

Supplement: Supplementary file 1 — Supplemental Figure1-8 and Supplemental Table 1 [file 41419_2023_5712_MOESM1_ESM.docx]
